# Genomic Insights into Antimicrobial Resistance and Plasmid-Mediated Dissemination in *Escherichia coli* and *Klebsiella pneumoniae* from Pediatric Outpatients with Acute Diarrhea

**DOI:** 10.3390/antibiotics15040331

**Published:** 2026-03-25

**Authors:** Linda Erlina, Fadilah Fadilah, Omnia Amir Osman Abdelrazig, Rafika Indah Paramita, Aisyah Fitriannisa Prawiningrum, Wahyu Dian Utari, Asmarinah, Yulia Rosa Saharman, Muzal Kadim, Badriul Hegar

**Affiliations:** 1 Doctoral Program in Biomedical Science, Faculty of Medicine, Universitas Indonesia, Jakarta 10430, Indonesia; linda.erlina22@ui.ac.id; 2 Department of Medical Chemistry, Faculty of Medicine, Universitas Indonesia, Jakarta 10430, Indonesia; rafikaindah@ui.ac.id (R.I.P.); wahyudianu0297@gmail.com (W.D.U.); 3 Master Program in Biomedical Science, Faculty of Medicine, Universitas Indonesia, Jakarta 10430, Indonesia; omniyajay@gmail.com; 4 Bioinformatics Core Facilities, Indonesian Medical Education and Research Institute (IMERI), Faculty of Medicine, Universitas Indonesia, Jakarta 10430, Indonesia; aisyahfitriannisa@gmail.com; 5 Department of Medical Biology, Faculty of Medicine, Universitas Indonesia, Jakarta 10430, Indonesia; asmarinah.si@ui.ac.id; 6 Department of Clinical Microbiology, Faculty of Medicine, Universitas Indonesia, Jakarta 10430, Indonesia; yulia.rosa01@ui.ac.id; 7 Dr. Cipto Mangunkusumo Hospital, Jakarta 10430, Indonesia; muzal.kadim@ui.ac.id (M.K.); hegar.syarif@ui.ac.id (B.H.); 8 Department of Child Health, Faculty of Medicine, Universitas Indonesia, Jakarta 10430, Indonesia

**Keywords:** antimicrobial resistance, multidrug resistance, plasmids, mobile genetic elements, *Escherichia coli*, *Klebsiella pneumoniae*

## Abstract

**Background**: Antimicrobial-resistant *Escherichia coli* and *Klebsiella pneumoniae* represent an increasing challenge in community-acquired pediatric diarrheal infections. Understanding the genomic basis and dissemination of resistance in outpatient settings is essential for guiding antimicrobial use. **Methods:** Eighteen Gram-negative isolates obtained from pediatric outpatients with acute diarrhea were analyzed using selective culture methods, antimicrobial susceptibility testing, and whole-genome sequencing. Multilocus sequence typing, serotyping, virulence profiling, antimicrobial resistance gene detection, plasmid replicon typing, mobile genetic element analysis, and core genome-based phylogenetic analysis were performed. Phenotypic resistance profiles were correlated with genomic resistance determinants. **Results:** *Klebsiella pneumoniae* (55.56%) and *Escherichia coli* (44.44%) were identified, with all isolates exhibiting putative multidrug resistance-associated genomic profiles. Extended-spectrum β-lactamase genes, particularly *blaCTX-M* variants, were strongly associated with resistance to third-generation cephalosporins. In contrast, fluoroquinolone resistance correlated with *gyrA* and *parC* mutations and plasmid-mediated *qnr* genes. Phylogenetic analysis revealed diverse lineages harboring resistance determinants. In silico plasmid analysis revealed that key resistance genes co-occurred with IncF-type plasmids and mobile genetic elements, including ISEcp1, IS26, and class 1 integrons, suggesting putative plasmid association rather than confirmed localization. **Conclusions:** These findings highlight the small scale of plasmid-mediated antimicrobial resistance among *E. coli* and *K. pneumoniae* causing pediatric community-acquired diarrhea. The integration of phenotypic and genomic analyses underscores the need for continuous resistance surveillance to support rational antibiotic use in outpatient settings.

## 1. Introduction

Diarrhea continues to be a critical public health issue in Indonesia, particularly among children under the age of five, where it ranks as a leading cause of morbidity and mortality. The burden of this illness is substantial, with an estimated 2.5 million cases annually in this age group and a national prevalence rate of 12.3% in 2022 [1]. Diarrhea in children can often lead to severe dehydration due to significant fluid and electrolyte loss, which can be life-threatening if not properly managed. The primary causative agents of diarrhea are diverse, including viruses, protozoa, and bacteria. Among these, Gram-negative bacteria such as *Escherichia coli*, *Shigella*, *Klebsiella pneumoniae*, *Salmonella*, and *Vibrio cholerae* are particularly notable [2]. These pathogens are often spread through contaminated food and water, as well as through contact with infected individuals or animals. *Escherichia coli*, a member of the Enterobacteriaceae family, is a common commensal in the human gut but can also cause severe, sometimes bloody diarrhea. Some strains of *E. coli*, along with other Gram-negative bacteria, have acquired mechanisms that enable them to resist multiple antibiotics, making infections difficult to treat and control [3].

In recent years, attention has increased toward antimicrobial resistance among community-acquired enteric pathogens, particularly *Escherichia coli* and *Klebsiella pneumoniae*. These organisms are not only frequent causes of pediatric diarrhea but also recognized reservoirs of antimicrobial resistance genes, including extended-spectrum β-lactamases (ESBLs) and plasmid-mediated quinolone resistance determinants. The dissemination of resistance via mobile genetic elements, such as plasmids, insertion sequences, and integrons, has facilitated the rapid spread of multidrug-resistant (MDR) phenotypes beyond hospital settings and into outpatient and community environments [4].

The particular concern is the growing evidence that resistance to commonly prescribed empirical antibiotics, including β-lactams, fluoroquinolones, and aminoglycosides, has become increasingly prevalent in pediatric populations. Resistance to these antibiotic classes compromises first-line treatment options for diarrheal infections and may lead to prolonged illness, increased healthcare utilization, and a greater risk of complications. In Gram-negative bacteria, resistance is often mediated by a combination of acquired resistance genes and chromosomal mutations affecting antibiotic targets, efflux regulation, and membrane permeability, underscoring the complexity of antimicrobial resistance mechanisms [5].

Whole genome sequencing offers a powerful framework for dissecting these resistance mechanisms at high resolution by simultaneously identifying resistance genes, mobile genetic elements, and phylogenetic relationships among isolates. Integrating WGS with phenotypic antimicrobial susceptibility testing enables a more comprehensive understanding of genotype–phenotype correlations and resistance dissemination pathways [6,7]. Such integrative approaches are particularly relevant in low- and middle-income countries, including Indonesia, where empirical antibiotic use remains common and genomic surveillance data for pediatric diarrheal pathogens are limited.

Therefore, in line with the global priority to combat antimicrobial resistance, this study focuses on the genomic characterization of antimicrobial-resistant *E. coli* and *K. pneumoniae* isolated from pediatric outpatients with acute diarrhea. By combining phenotypic susceptibility testing with whole-genome-based analysis of resistance determinants and mobile genetic elements, this work aims to provide evidence relevant to antimicrobial stewardship and resistance surveillance in community settings.

## 2. Materials and Methods

### 2.1. Fecal Sample Collection

Children aged 2–5 years with clinical acute diarrhea were recruited using consecutive sampling. Inclusion criteria were: (1) children with clinical acute diarrhea aged 2–5 years, and (2) parental consent. The exclusion criterion was an insufficient fecal sample (<3 g). Primary data were collected through parent interviews and physical examinations. Fecal samples were collected and transported to the FKUI Microbiology Laboratory within 24 h.

A total of 18 subjects who met the inclusion criteria and agreed to sign the informed consent form (ICF) were enrolled as subjects, with a sample size calculation formula [8]:

$$n\;=\;\frac{{Za}^{2}\times P\times (1-P)}{d^{2}}=\;\frac{1.96^{2\;}\times \;0.0123\;\times \;(1-0.123)\;}{{\left(0.15\right)}^{2}}=\;18\;\mathrm{samples}$$

n = sample size

Za = Z statistic for a level of confidence (1.96 for 95% confidence level)

P = proportion of cases based on literature (12.3%)

(According to the Indonesian Ministry of Health, 2022) [1]

d = Precision 0.15 (15%)

Research was approved by the Ethics Committee of the Faculty of Medicine, University of Indonesia, and by the Ethics Committee of Dr. Cipto Mangunkusumo National General Hospital. The approval number for this study was ND-53/UN2.F1/ETIK/PPM.00.02/2024 (approved on 19 January 2024).

### 2.2. Gram-Negative Bacteria Culture, DNA Isolation, and Whole Genome Sequencing

Samples were inoculated onto selective agar plates (XLD, EMB, Salmonella-Shigella, MacConkey, and TCBS) using the semiquantitative streak plate method and incubated for 24 h. After incubation, colonies showing morphology consistent with Gram-negative enteric bacteria were selected for further identification. For each sample, the dominant colony morphology was subcultured to obtain a pure isolate, which was subsequently identified using the VITEK^®^ 2 Compact system (bioMérieux, Marcy-l’Étoile, France). This method was used to obtain a representative, clinically relevant isolate from each specimen, consistent with routine clinical microbiology practice, in which the predominant colony recovered from a culture is selected for downstream phenotypic or genomic analysis.

DNA was extracted using a QiAmp DNA Mini Kit (Qiagen, Hilden, Germany) following the manufacturer’s protocol [9]. DNA purity and concentration were assessed using a Nanodrop spectrophotometer and Qubit 4.0 fluorometer (Thermo Fisher Scientific, Waltham, MA, USA). All samples passed quality control, with DNA purity ratios (A260/280) ranging from 1.8 to 2.00 and DNA concentrations above 10 ng/L. The DNA purity ratio (OD260/280) ranges from 1.77 to 2.00, and DNA concentration from 18.18 to 84.007 ng/μL. Library preparation was performed using the Nextera Illumina tagmentation kit (Illumina, San Diego, CA, USA). Sequencing was performed using paired-end reads on the Illumina MiSeq platform (Illumina, San Diego, CA, USA) [10].

### 2.3. Antimicrobial Susceptibility Testing

Antimicrobial susceptibility testing was performed using the VITEK^®^ 2 automated system (bioMérieux, Marcy-l’Étoile, France) according to the manufacturer’s instructions. The representative isolates obtained from each sample were subcultured on non-selective agar, and standardized suspensions were prepared in sterile saline to a turbidity equivalent to 0.5 McFarland, as required by the VITEK^®^ 2 system. Susceptibility testing was conducted using VITEK^®^ 2 AST cards appropriate for Enterobacterales. The system automatically measured bacterial growth kinetics and assigned minimum inhibitory concentration (MIC) values. MIC interpretations (susceptible, intermediate, or resistant) were determined using Clinical and Laboratory Standards Institute (CLSI) breakpoints embedded in the VITEK^®^ 2 software version 10 (CLSI M100) [11], edition current at the time of testing). Quality control procedures were performed in accordance with CLSI recommendations using *Escherichia coli* ATCC 25922 and *Klebsiella pneumoniae* ATCC 700603. All quality control results were within acceptable ranges.

### 2.4. Whole Genome Analysis (Serotype, Resistance Gene, and Virulence Factor)

The quality of paired-end reads from each Gram-negative bacterial isolate was assessed using FastQC version 0.11.4 [12]. Before assembly, low-quality reads (below Q20) were filtered using Trimmomatic, and high-quality reads were further processed (Table S1) [13]. The analyses carried out for this research included Multilocus Sequence Typing (MLST) using version 2.0.4, with the database updated to version 2.0.1 on 1 July 2022 [14]. Serotyping was performed with SeroTypeFinder version 2.0.1, utilizing the database version 2.0.0 from 1 January 2023 [15]. Virulence genes were identified using VirulenceFinder version 2.0, which had a database version from 31 May 2022 [16,17]. Antimicrobial resistance (AMR) genes were detected using ResFinder version 3.2, with the database updated to version from 1 June 2022 [18]. VirulenceFinder identified 29 virulence genes, of which 12 are ExPEC-associated, 16 are EAEC-associated, and 1 is EPEC-associated, irrespective of pathotype. For the detection standard parameters were set as follows: VirulenceFinder and SerotypeFinder, 85% sequence identity and 60% sequence coverage; ResFinder, 90% sequence identity and 60% sequence coverage; MLST, using the seven loci (*gyrB*, *adk*, *icd*, *purA*, *recA*, *fumC*, and *mdh*) scheme [15].

### 2.5. Core Genome Phylogenetic Analysis

Core genome-based phylogenetic analysis was performed to assess the genetic relatedness and population structure of *Escherichia coli* and *Klebsiella pneumoniae* isolates. Whole-genome sequences were analyzed using a single-nucleotide polymorphism (SNP) based or core genome multilocus sequence typing (cgMLST) approach. Core genome alignment using Roary 3.13.0 generated.aln files, and phylogenetic inference was performed using the phangorn 2.12.1 package in R version 4.5.0 [19,20]. Maximum-likelihood trees were reconstructed under the GTR+Γ+I model, with 1,000 bootstrap replicates. The trees were mid-rooted and visualized using ggtree [21]. Phylogenetic trees were constructed from core genome alignments and visualized to assess clustering patterns. Annotation layers were applied to indicate bacterial species, sequence types (STs), virulence pathotypes, and multidrug resistance (MDR) status.

### 2.6. Plasmid Replicon Typing and Mobile Genetic Element Analysis

Major plasmid groups, including IncF, IncHI, IncX, and IncR, were screened in all isolates. Mobile genetic elements (MGEs), including class 1 integrons and insertion sequences such as ISEcp1 and IS26. The co-occurrence of plasmid replicons, MGEs, and antimicrobial resistance genes was assessed to evaluate potential mechanisms of horizontal gene transfer. Determination of plasmid replicon type was performed using PlasmidFinder v2.1 [22]. Analysis was performed using default CGE parameters, with a minimum sequence identity threshold of ≥95% and minimum coverage of ≥60%. Mobile genetic elements (MGEs) were identified using complementary in silico tools and databases. Insertion sequences were detected by sequence similarity searches against the ISfinder database using ISEScan v1.7.3 [23]. Integron-associated elements were determined using IntegronFinder v2.0 to detect the integrase gene (*intI*), the attC recombination site, and conserved segments characteristic of class 1 integrons [24]. Putative predictions of co-localization of antimicrobial resistance genes (ARGs) with plasmid replicons or MGEs were assessed at the contig level. Relatedness is inferred when ARGs and replicon markers or MGEs are present on the same or adjacent assembled contigs, providing supporting but not definitive evidence of plasmid-mediated spread.

## 3. Results

### 3.1. Gram-Negative Bacteria Culture

To ensure and validate the colony’s identity, growth, and purity, colony morphology (macroscopic) phenotypic characterization from various media such as Xylose Lysine Deoxycholate (XLD), Eosin Methylene Blue (EMB), Selenite broth, Thiosulfate Citrate Bile Salts Sucrose (TCBS), and MacConkey media were evaluated with semiquantitative interpretation (+1 until +4) (Table 1).

**Figure 1:**
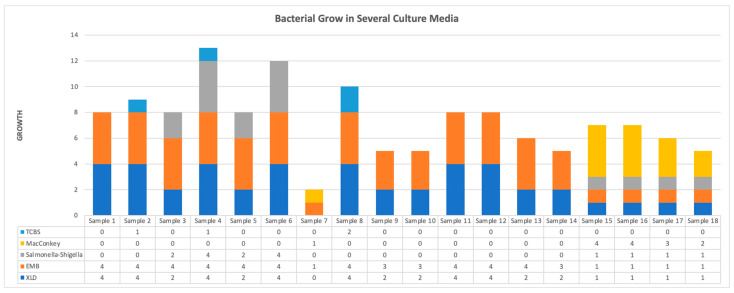
Bacteria grow in several culture media.

Analysis of fecal samples using various culture media revealed distinct microbial growth patterns and semiquantitative results. On Xylose Lysine Deoxycholate (XLD) media, the predominant observation was yellowish colonies, suggesting xylose fermentation, which accounted for 88.88% of the samples and was interpreted as +1 (samples 15, 16, 17, and 18). A minority of samples displayed more substantial growth, with interpretations of +2 and +4 distributed among samples 3, 5, 9, 10, 13, and 14 for +2, and samples 1, 2, 6, 8, 11, and 12 for +4. Eosin Methylene Blue (EMB) media consistently showed brownish colonies with a +4 interpretation across samples 1, 2, 5, 6, 12, and 13. Additionally, variations in colony appearance were noted, including metallic green colonies representing lactose-fermenting coliforms for the presence of *Escherichia coli,* with a +4 interpretation, and a single sample (16) exhibiting black colonies with a +1 interpretation. Samples showed varying growth intensities, including +3 in samples 9, 10, and 14, and a single instance of metallic brownish with a +4 interpretation in sample 11. One sample (15) displayed smooth colonies with a +1 interpretation. All other samples (3–18) showed turbidity. On Salmonella-Shigella media, pink colonies with a +1 interpretation were observed in samples 16, 17, and 18, representing 38.89% of the samples. Other samples showed varied growth with interpretations of +2 and +4. Thiosulfate Citrate Bile Salts Sucrose (TCBS) media showed yellowish colonies with a +1 interpretation in 16.67% of samples (2 and 4), while sample 8 displayed greenish colonies with a +2 interpretation. Lastly, MacConkey media revealed pinkish colonies with interpretations ranging from +1 to +4 across different samples (Figure 1).

As shown in Figure 1, seven samples (1, 9, 10, 11, 12, 13, and 14) have at least 2 isolates per sample identified in the cultured results using XLD and EMB media. Four samples (samples 3, 5, and 6) have at least 3 isolates per sample identified in cultured results using XLD, EMB, Salmonella, and Shigella media. Four samples (15–18) have at least 4 isolates per sample identified in cultured results using XLD, EMB, Salmonella, Shigella, and MacConkey media. Samples 2 and 8 have at least 3 isolates per sample identified in cultured results with XLD, EMB, and TCBS media. Sample 4 has at least 4 isolates per sample identified in cultured results on XLD, EMB, Salmonella, Shigella, and TCBS media. Sample 7 has at least 2 isolates per sample identified in cultured results with EMB and MacConkey media. Further details on culture results for all samples in different media are shown in Supplementary Materials Figures S1–S5.

**Figure 2:**
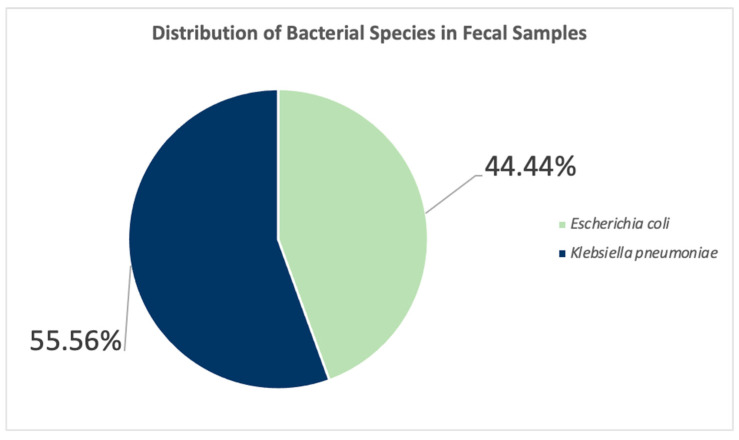
Distribution of Bacterial Species in Fecal Samples.

In the analysis of 18 distinct samples, two bacterial strains were identified: *Klebsiella pneumoniae* and *Escherichia coli*. *Klebsiella pneumoniae* was the predominant bacterium, being present in 55.56% of the samples (specifically samples 1, 2, 4, 5, 6, 10, 11, 12, 13, and 17). On the other hand, *Escherichia coli* was found in 44.44% of the samples, represented by samples 3, 7, 8, 9, 14, 15, 16, and 18. Notably, *Vibrio* culture was negative for all samples tested (Figure 2).

### 3.2. Multilocus Sequence Typing and Serotyping

After trimming, all FASTQ samples have high-quality reads (above Q30) with a percentage of 85–94% (Table S1). This high-quality read set will indicate high confidence in MLST results. From the total of 18 bacterial samples, two types of bacteria were identified: *Klebsiella pneumoniae* and *E.coli* (refer to Table 2). Ten samples (55.56%) belonged to *Klebsiella pneumoniae*, featuring a diverse range of Sequence Types (STs), serotypes, and capsular types, with multiple samples exhibiting phylotypes such as K1, K2, K17, K24, and K5. For example, both Sample 1 and Sample 13 were of the K2 phylotype, despite having different STs and serotypes: ST-231 with serotype KL51/O1 for Sample 1 and ST-65 with serotype KL2/O1 for Sample 13. Similarly, Sample 2 and Sample 6 shared the K17 phylotype, both being ST-101 but with different serotypes: KL17/O1 for Sample 2 and KL2/O1 for Sample 6. In contrast, 8 samples (44.44%) were identified as *E. coli*. These samples also demonstrated variability in their Sequence Types, serotypes, and capsular types, with notable phylotypes including A, B2, D, and B1. For instance, both Sample 3 and Sample 9 had the ST-10 sequence type and belonged to phylotype A, but differed in serotype: O153 for Sample 3 and O169 for Sample 9. Additionally, Samples 7 and 15, despite having different STs, both belonged to phylotype D with serotypes O7 and O15, respectively.

### 3.3. Virulence Genes

A total of eight *E. coli* isolates were analyzed (Table S2), all of which harbored a diverse array of virulence genes associated with various pathogenic mechanisms. The most prevalent gene was *fimH*, found in 6 isolates (75%), underscoring its significant role within the Adhesins group. Additionally, five isolates (62.5%) carried genes for the Iron Binding Protein group, indicating the importance of this group in the overall virulence profile. Notably, *irp1* and *irp2* were detected in 4 samples (50%), highlighting their co-occurrence and significance within the Siderophores and Iron Transport Protein groups. In the Miscellaneous group, all eight isolates (100%) contained virulence genes, reflecting the diversity of this category. Other prevalent genes, such as *irp1*, *irp2*, *yehD*, *iutA*, *fyuA*, *traT*, *nlpI*, *csgA*, *AslA*, *clbA*, *clbB*, *hlyE*, and *AstA*, had occurrence rates ranging from 25% to 62.5%. Genes with moderate prevalence (12.5% to 37.5%) included *Aap*, *afaA*, *afaB*, *air*, *capU*, *eilA*, *mrkA*, *fdeC*, *gad*, *terC*, *yehA*, *yehB*, *yehC*, *hlyA*, *hlyB*, *hlyC*, *hlyD*, *sat*, *iucC*, and *uge*.

The virulence gene distribution in *E. coli* revealed that Extraintestinal pathogenic *E. coli* (ExPEC) was the most prevalent, accounting for 79.59% of strains. ExPEC harbored numerous virulence-associated genes, including iron acquisition systems (*irp1*, *irp2*, *ybtSXQPA*, *fyuA*, *iutA*, *ybt*), serum resistance factors (*traT*, *iss*), heme receptors (*chuA*), tellurite resistance (*terC*), and various toxins, adhesins, and regulators. Uropathogenic *E. coli* (UPEC), comprising 16.33% of strains, possessed eight virulence genes overlapping with the ExPEC repertoire. Enteroaggregative *E. coli* (EAEC) and enterotoxigenic *E. coli* (ETEC) accounted for 4.08% of strains each, with specific virulence genes associated with their pathogenicity. Diffusely adherent *E. coli* (DAEC) accounted for 6.12% of strains, while Avian pathogenic *E. coli* (APEC) was the least prevalent at 4.08%.

In *Klebsiella pneumoniae*, all ten isolates examined exhibited a diverse array of virulence genes, illustrating the complexity of *Klebsiella* virulence factors. The Adhesins group was particularly prominent, with seven isolates (70%) carrying one or more genes from this category. *FimH-1* was notably prevalent, found in five isolates (50%), signifying its dominant role within the Adhesins group. Regarding iron acquisition, which is critical for bacterial survival and virulence, genes related to the Iron Uptake System, such as *fyuA*, were highlighted. *fyuA*, a gene encoding yersiniabactin, was detected in 4 isolates (40%). The genes *irp1* and *irp2*, involved in siderophore production, were present in three isolates (30%), underscoring their importance in the Siderophores and Iron Transport Protein groups. Capsule Synthesis Regulator genes, such as *RmpA* and *RmpA2*, were detected in 5 isolates (50%), suggesting their role in evading host immune responses. The Miscellaneous group, encompassing a broader range of virulence factors, was represented in 8 isolates (80%), with genes such as *magA*, *hlyA*, and *uge* contributing to their virulence. Moderately prevalent genes included *mrkD*, present in four isolates (40%), and *clbA* and *clbB*, each found in three samples (30%).

Analysis of 34 virulence genes in *Klebsiella pneumoniae* highlighted differences between hypervirulent *K. pneumoniae* (hvKp) and classic *K. pneumoniae* (cKp) pathotypes. Genes associated with hvKp, comprising 61.76% of the total genes studied, included those involved in siderophore biosynthesis and transport (*fyuA*, *iutA*, *iucA*, *iroB*, *ybtS*), capsule biosynthesis (*capU*, *kpsS*, *kpsE*, *kpsF*), and mucoid phenotype regulation (*RmpA2*, *RmpA*, *rmpC*). These genes enhance hvKp strains’ ability to evade host immune responses and cause severe infections. Conversely, genes more frequently associated with cKp included those involved in type 1 fimbrial protein production (*fimH-1*, *fimA*, *fimE*), outer membrane proteins (*ompA*, *pilA*), and various fimbrial assembly proteins (*mrkD*, *mrkB*, *mrkE*, *mrkA*).

### 3.4. Antimicrobial Resistance

The occurrence of antibiotic resistance genes among *K. pneumoniae* isolates is substantial. Fluoroquinolone resistance was highly prevalent, with 90% of the isolates carrying the *OqxA* gene and 60% harboring *OqxB*. Among fosfomycin-resistant isolates, 80% carried *fosA*. All *K. pneumoniae* isolates exhibited genes encoding aminoglycoside resistance enzymes, with *aac(3)-IId*, *aadA1*, and *aph(3″)-Ib* each found in 30% of the isolates. Anti-beta-lactam resistance genes were present in all *K. pneumoniae* isolates, with *blaCTX-M-15* and *blaTEM-209* each found in 40% of the isolates, followed by *blaOXA-232* and *blaTEM-1B* each in 30%. Chloramphenicol resistance genes, *OqxA* and *OqxB*, were each found in 70% of the isolates. Trimethoprim resistance genes were detected in 40% of the isolates, with *dfrA14* being the most common (20%). Sulfonamide resistance genes *sul2* and *sul1* were found in 40% and 20% of the isolates, respectively. Tetracycline resistance was moderate, with 20% of the isolates carrying *tet(A)*. Notably, none of the *K. pneumoniae* isolates carried macrolide resistance genes.

In *E. coli* isolates, all carried genes encoding aminoglycoside resistance, including *aac(3)-IId*, *aac(6′)-Ib-cr*, *aadA2*, *aadA5*, and *aph(3”)-Ib*, with varying prevalences. All *E. coli* isolates also showed anti-beta-lactam resistance genes, including *blaCTX-M-27*, *blaCTX-M-10*, *blaOXA-15*, *blaOXA-1*, and *blaTEM-1B*. Trimethoprim resistance genes were present in 37.5% of the isolates, with *dfrA14*, *dfrA17*, and *dfrA12* each detected in 12.5%. Chloramphenicol resistance gene *catB3* was present in 25% of the isolates. Fluoroquinolone resistance genes were also prevalent, with 75% of the isolates carrying *OqxA*, 37.5% carrying *OqxB*, and 12.5% carrying *qnrS1*. Fosfomycin resistance was noted in 25% of the isolates with *fosA6*. Sulfonamide resistance genes *sul1* and *sul2* were found in 25% and 12.5% of the isolates, respectively. Macrolide resistance gene *mph(A)* was present in 37.5% of the isolates. Tetracycline resistance genes were found in 50% of the isolates, with 37.5% carrying *tet(A)* and 12.5% carrying *tet(B)*.

Comparing susceptibility test results and genomic profiles provides detailed insights into antibiotic resistance patterns in bacterial samples. This analysis is particularly useful for correlating specific genetic markers with observed phenotypic resistance to various antibiotics. Below is an elaboration of the findings from Table S3, which examines samples of *Klebsiella pneumoniae* (KP) and *Escherichia coli* (*E. coli*). For *Klebsiella pneumoniae* (KP), Sample 1 demonstrated resistance to Ampicillin, Ceftazidime, Ciprofloxacin, and Piperacillin-Tazobactam, while it was sensitive to Trimethoprim. The genomic profile revealed resistance genes corresponding to these antibiotics: *blaCTX-M-15* for Ampicillin and Ceftazidime, and *OqxA* for Ciprofloxacin. The resistance to Piperacillin-Tazobactam was also attributed to *blaCTX-M-15*. Sample 2 showed resistance to all tested antibiotics, with genomic markers such as *blaSHV-5* for Ampicillin, *OqxAB* for Trimethoprim, and *blaCTX-M-15* for Ceftazidime and Ciprofloxacin, confirming the phenotypic resistance.

Sample 4 exhibited resistance to Ampicillin but was sensitive to the other antibiotics tested. Its genomic profile indicated the presence of *blaOXA-232* for Ampicillin resistance and *OqxAB* for Trimethoprim, which aligns with the phenotypic resistance. Sample 5 was resistant to Ampicillin, Trimethoprim, and Ciprofloxacin, with resistance genes *blaSHV62* for Ampicillin, *OqxAB* for Trimethoprim, and *OqxA* for Ciprofloxacin, confirming the phenotypic results. Sample 6 showed resistance to Ampicillin but was sensitive to the other antibiotics. The genomic data indicated resistance genes, such as *blaCTX-M-15* for Ampicillin and *OqxAB* for Trimethoprim, which correlated well with the susceptibility results. Sample 10 was sensitive to all antibiotics except Trimethoprim, where the presence of *OqxAB* was noted in the genomic profile. Sample 11 was resistant to Ampicillin but sensitive to other antibiotics. Its genomic profile showed the *dfrA14* gene for Trimethoprim resistance and *OqxB* for Ciprofloxacin, indicating potential resistance mechanisms. Sample 12 was resistant to Ampicillin, harboring *blaOXA-232* and *OqxAB*, while remaining sensitive to other antibiotics. For *Escherichia coli* (*E. coli*), Sample 3 showed resistance to Ampicillin and Trimethoprim, with corresponding resistance genes *blaCTX-M-27* for Ampicillin and *OqxAB* for Trimethoprim.

Sample 7 demonstrated resistance to Ampicillin and Ciprofloxacin, with genomic markers *blaOXA-10* and *qnrS1*, respectively, matching the phenotypic resistance. Sample 8 was resistant to Ampicillin, Trimethoprim, and Ciprofloxacin. The genomic profile indicated the presence of *blaCTX-M-15* and *OqxAB* genes for these antibiotics, confirming the phenotypic results. Sample 9 was sensitive to all antibiotics, except for those carrying *blaCTX-M-15* and *OqxAB*, suggesting potential resistance not detected phenotypically. Sample 14 was resistant to Trimethoprim, with the *dfrA14* gene noted in the genomic profile, aligning with the susceptibility test. Sample 15 showed resistance to Ampicillin and Trimethoprim, with corresponding resistance genes *OqxAB*. Sample 16 was resistant to Ampicillin, with the *OqxAB* gene detected in its genomic profile, consistent with the phenotypic resistance. Lastly, Sample 18 was resistant to Ampicillin, with the *dfrA12* gene identified in the genomic data, confirming the observed phenotypic resistance.

### 3.5. Amino Acid Mutations Related to Antimicrobial Resistance Genes

**Figure 3:**
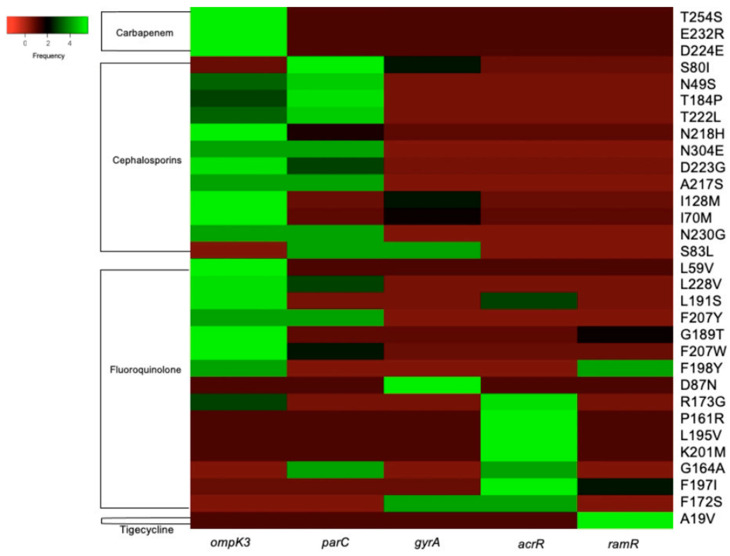
Heatmap illustrating the resistance profiles associated with various mutations.

The heatmap showing resistance profiles of bacterial mutations to antibiotics such as carbapenems, cephalosporins, fluoroquinolones, and tigecycline is shown in Figure 3.

The heatmap (Figure 3) shows the distribution of amino acid substitutions across several key genes associated with different antimicrobial resistances. The associated genes, such as *ompK3*, *parC*, *gyrA*, *acrR*, and *ramR*, encode several antimicrobial resistance mechanisms, including membrane permeability, target modification, and efflux regulation. Mutations in the *ompK3* gene, which modulate β-lactam entry, are associated with carbapenem and cephalosporin resistance by disrupting an outer membrane porin mechanism. Some high-frequency substitutions at several amino acid positions, such as T254S, E232R, and D224E, were reported to decrease membrane permeability, a major contributor to reduced susceptibility.

Recurrent mutations in *gyrA* and *parC*, particularly substitutions in the canonical quinolone resistance-determining region (QRDR), include S83L and D87N in *gyrA* and several changes in *parC*. This mutation is known to disrupt the binding of fluoroquinolones to DNA gyrase and topoisomerase IV. High-frequency mutations in the *acrR* and *ramR* genes, which encode negative regulators of the AcrAB-TolC efflux system, have been detected in various classes of antibiotics, particularly fluoroquinolones and tigecycline. High-frequency substitutions (R173G, P161R, L195V, K201M) derepressed efflux pump expression, conferring a multidrug resistance phenotype rather than class-specific resistance.

Mutations in the porin-encoding gene (*ompK3*), target site mutations (*gyrA*/*parC*), and changes in the efflux regulator (*acrR*/*ramR*) suggest multidrug resistance in children’s diarrhea samples. Overall, analyzing the frequency of mutations in key associated genes and antimicrobial types will help understand that a single mutation does not drive antimicrobial resistance; instead, it requires the accumulation of mutations across several key associated genes.

### 3.6. Correlation Between Phenotypic Antimicrobial Susceptibility and Genomic Resistance Determinants

To assess the association between phenotypic antimicrobial resistance and underlying genomic determinants, antimicrobial susceptibility testing (AST) results were correlated with the presence or absence of antimicrobial resistance genes (ARGs) and resistance-associated chromosomal mutations. The analysis compared resistant and non-resistant isolates across major antibiotic classes to determine genotype–phenotype concordance, as shown in Table 3.

Table 3 summarizes the small-scale association between antimicrobial resistance phenotypes and the presence of resistance genes or amino acid substitutions. Resistance rates are expressed as percentages of resistant isolates among gene or mutation-positive strains. Statistical significance was assessed using Fisher’s exact test, with *p*-values indicating the strength of the association between genotype and phenotype.

### 3.7. Phylogenetic Analysis and Population Structure

**Figure 4:**
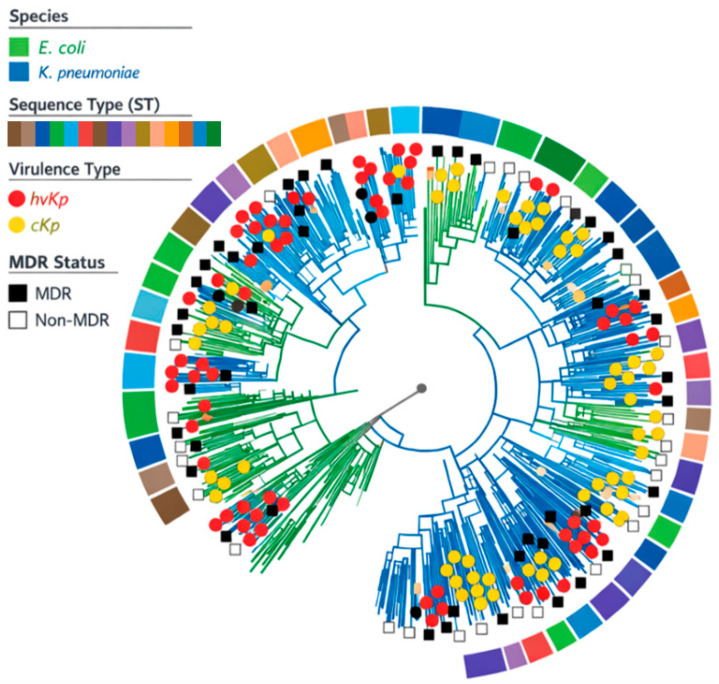
Core genome phylogenetic tree of *Escherichia coli* and *Klebsiella pneumoniae* isolates.

Core genome–based phylogenetic analysis was performed to investigate the population structure and genetic relatedness of *Escherichia coli* and *Klebsiella pneumoniae* isolates. Phylogenetic clustering revealed distinct lineages corresponding to bacterial species and sequence types. Several clusters were enriched with hypervirulent *K. pneumoniae* (hvKp) isolates, while others predominantly comprised classical *K. pneumoniae* (cKp) or *E. coli*. Multidrug-resistant isolates were distributed across multiple lineages, with certain clusters showing co-occurrence of hypervirulence and MDR profiles. Figure 3 was constructed based on core genome SNP/cgMLST analysis. Branch colors indicate bacterial species (*E. coli* and *K. pneumoniae*), while annotation layers denote sequence type (ST), virulence classification (hvKp vs. cKp), and multidrug resistance (MDR) status. The phylogeny illustrates lineage-specific clustering and the distribution of virulence and resistance traits among isolates (Figure 4).

### 3.8. Plasmid Replicon Types and Mobile Genetic Elements

Plasmid replicon typing and analysis of mobile genetic elements (MGEs) were performed to characterize the genetic platforms associated with antimicrobial resistance genes (ARGs) in *Escherichia coli* and *Klebsiella pneumoniae* isolates. The distribution of plasmid incompatibility groups and MGEs was examined to assess their association with key resistance determinants, as shown in Table 4.

Plasmid replicon analysis revealed that resistance genes were predominantly associated with IncF-type plasmids, followed by IncHI, IncX, and IncR replicons. Extended-spectrum β-lactamase (ESBL) genes, particularly *blaCTX-M* variants, were most frequently detected on IncF plasmids. Carbapenemase genes and fosfomycin resistance genes were also identified on IncHI and IncR plasmids in several isolates. Mobile genetic element analysis demonstrated the widespread presence of class 1 integrons among multidrug-resistant isolates. Insertion sequences, particularly ISEcp1 and IS26, were frequently detected in the vicinity of ESBL genes, suggesting a role in gene mobilization and dissemination. The co-occurrence of ARGs, plasmid replicons, and MGEs suggests active horizontal gene transfer contributing to the spread of antimicrobial resistance.

## 4. Discussion

In this study, various selective and differential media were employed to isolate and identify Gram-negative bacteria from fecal samples. Xylose Lysine Deoxycholate (XLD) agar predominantly yielded yellowish colonies, indicating lactose-fermenting Enterobacterales, including *K. pneumoniae*. Eosin Methylene Blue (EMB) agar effectively differentiated lactose fermenters such as *Escherichia coli*, characterized by metallic green colonies, from non-lactose fermenters, including *K. pneumoniae*, which appeared as brownish colonies. These findings are consistent with standard microbiological identification approaches for enteric pathogens [25]. The semiquantitative variation in colony density across media reflects heterogeneous bacterial loads, a common feature of pediatric diarrheal samples with mixed microbiota. In cases of diarrhea, these two bacterial strains overgrow, contributing to dysbiosis. The selective and differential media for culturing bacteria targeted Gram-negative enteric pathogens; other pathogens, such as viruses, obligate anaerobes, and toxin-mediated agents, were not covered under aerobic culture conditions [26].

Our genomic characterization of *K. pneumoniae* revealed substantial concordance with previous reports on high-risk and hypervirulent lineages. The identification of ST-15, KL38/O1, K24 (capsular type) aligns with studies describing this clone as a globally disseminated lineage frequently associated with virulence determinants and multidrug resistance [27]. Prior investigations have demonstrated that ST-15 isolates often harbor genes such as *rmpA*, *magA*, *entB*, *ybtS*, and *iutA*, contributing to enhanced capsule production, iron acquisition, and immune evasion, while simultaneously exhibiting resistance to multiple antimicrobial classes, including carbapenems in some regions [27]. The presence of ST-101 and ST-4004 in this study further supports their role as genetically diverse and clinically relevant clones, frequently associated with hospital and community-acquired infections and high-risk resistance profiles [28,29].

Among *E. coli* isolates, ST-10 O153:H10 has been reported in previous studies and is described as a hybrid EPEC/ExPEC strain capable of causing both intestinal and extraintestinal infections [30]. ST-10 is widely distributed across hosts and ecological niches. It is often associated with a broad virulence gene repertoire, including eae variants and iron acquisition systems, underscoring its adaptive potential and public health relevance [31].

The predominance of hypervirulent *K. pneumoniae* (hvKP) in this cohort is noteworthy, with 61.76% of virulence genes corresponding to hvKP-associated determinants. Genes involved in siderophore biosynthesis (*fyuA*, *iutA*) and capsule formation (*kpsS*, *kpsE*) were frequently detected, reinforcing the established role of these factors in enhancing bacterial fitness and pathogenicity [32]. Consistent with global epidemiological trends, capsular types K1 and K2 were prevalent, serotypes known for their enhanced resistance to phagocytosis and serum killing [33]. In contrast, ExPEC-associated *E. coli* strains accounted for the majority of virulence profiles, characterized by diverse adhesins, iron uptake systems, serum resistance factors, and toxins, reflecting their capacity to cause invasive disease beyond the gastrointestinal tract [34]. The lower prevalence of EAEC, ETEC, DAEC, and APEC pathotypes aligns with previous pediatric diarrheal studies that found ExPEC and EPEC predominate in small-scale community settings [35].

Importantly, integrating phenotypic antimicrobial susceptibility testing with genomic resistance profiling revealed strong genotype–phenotype concordance. The widespread presence of *blaCTX-M-15* was significantly associated with resistance to third-generation cephalosporins, reinforcing its central role in extended-spectrum β-lactamase (ESBL) mediated resistance [36]. This finding is also supported by several previous studies, for example, J.J. van Aartsen et al. (2019) [37]. In this study, the epidemiology of pediatric gastrointestinal colonization by extended-spectrum cephalosporin-resistant *Escherichia coli* and *Klebsiella pneumoniae* isolates in north-west Cambodia was found to be common in Southeast Asia, as evidenced by the *blaCTX-M* gene marker [37]. Nuru Letara et al. (2021) reported a high prevalence of Extended-Spectrum Beta-Lactamase (ESBL) producing *Escherichia coli* and *Klebsiella pneumoniae* carriage and infection among pediatric patients in Tanzania, due to the high use of antibiotics [38].

Fluoroquinolone resistance correlated with both plasmid-mediated determinants (qnrS1) and chromosomal mutations within the quinolone resistance-determining regions of *gyrA* and *parC*, consistent with established resistance mechanisms described in Enterobacterales [39]. These findings highlight the multifactorial nature of resistance, driven by the interplay between acquired genes and chromosomal mutations.

Phylogenetic analysis further demonstrated that multidrug resistance and hypervirulence were distributed across multiple genetic lineages rather than confined to a single clonal cluster. This observation suggests ongoing dissemination of resistance and virulence determinants through horizontal gene transfer rather than solely through clonal expansion, a pattern increasingly reported in community-acquired infections [40]. The detection of MDR profiles among outpatient isolates underscores the shifting epidemiology of antimicrobial resistance from hospital to community settings at the pilot scale.

Putative plasmid and mobile genetic element analyses based on in silico prediction provided additional insights into the mechanisms underlying resistance dissemination. The predominance of IncF-type plasmids carrying *blaCTX-M* genes is consistent with numerous studies that identify IncF plasmids as key vectors in the global spread of ESBL-producing Enterobacterales [41]. The frequent association of ESBL genes with insertion sequences such as ISEcp1 and IS26, as well as class 1 integrons, underscores their role in mobilizing and stabilizing resistance genes within bacterial populations [42]. These mobile elements facilitate rapid adaptation to antimicrobial pressure and contribute to the persistence of resistance in community reservoirs. Since this study uses short-read sequencing, the entire plasmid assembly is frequently reported as “ND” due to fragmented replicon-repetitive elements, shared backbone regions across different incompatibility groups, and the presence of insertion sequences and integrons, which can result in fragmented contigs lacking complete replicon marker genes. Consequently, plasmid replicons may not be confidently assigned, even when resistance genes are plasmid-borne.

The amino acid substitutions identified in *K. pneumoniae*, particularly within *gyrA*, *parC*, and *ompK36*, further elucidate resistance mechanisms at the protein level. Mutations such as S83L and D87N in *gyrA* are well-established contributors to high-level fluoroquinolone resistance. At the same time, alterations in *parC* and porin proteins reduce antibiotic influx and enhance resistance to cephalosporins and carbapenems [43,44]. Regulatory mutations affecting efflux systems, including those involving *AcrR* and *RamR*, may further potentiate multidrug resistance, as reported in previous functional studies [45].

Collectively, these findings highlight the convergence of potentially hypervirulent, multidrug-resistant, and plasmid-mediated gene transfer among *E. coli* and *K. pneumoniae* isolates recovered from pediatric outpatients with acute diarrhea. The previous review studies by Miran Tang et al. (2020) [46], Gabriele Arcari and Alessandra Carattoli (2023) [47], and Dania Al Ismail et al. (2025) [48] also define hypervirulent clones of *K. pneumoniae* in Eastern Asia with specific STs, such as ST-23, ST-65, and ST-86. Relevant to this study, ST-23 and ST-65 are predicted as hypervirulent clones from samples 12 and 13, identified as hvKp [46,47,48].

The presence of such high-risk genomic features in small-scale, community-managed cases underscores the need for continuous genomic surveillance and underscores the importance of antimicrobial stewardship strategies to mitigate the spread of resistant pathogens in pediatric populations. These findings underscore the selective pressure exerted by commonly used antibiotics in pediatric outpatient settings and highlight the need for resistance-informed empirical therapy.

Some limitations of this study include the use of short-read whole-genome sequencing, which limits the ability to fully and definitively decipher plasmid structure and to localize antimicrobial resistance and virulence genes. By implementing in silico analysis, putative plasmid replicon assignment and ARG-plasmid association were inferred to identify co-localization and the presence of mobile genetic elements, rather than experimentally confirming plasmid reconstruction. Long-read or hybrid sequencing is necessary to address these associations fully.

Second, the culture-based approach used in this study selectively isolates fast-growing aerobic Gram-negative bacteria and does not cover the full spectrum of enteric pathogens, including viruses, obligate anaerobes, or difficult-to-culture organisms. These findings reflect the genomic characteristics of culturable *E. coli* and *K. pneumoniae* isolates; whole shotgun sequencing is strongly recommended to find the representation of the microbial causes of diarrhea.

## 5. Conclusions

This study demonstrates that *Escherichia coli* and *Klebsiella pneumoniae* isolated from pediatric outpatients with acute diarrhea harbor diverse and clinically relevant antimicrobial resistance determinants. The relationship between phenotypic resistance and genomic findings highlights the contribution of extended-spectrum β-lactamases, plasmid-mediated quinolone resistance genes, and resistance-associated chromosomal mutations to multidrug-resistant phenotypes. The predominance of resistance genes located on IncF-type plasmids and their frequent association with mobile genetic elements underscore the role of horizontal gene transfer in the dissemination of antimicrobial resistance in small-scale community settings. These findings emphasize that multidrug-resistant bacteria are not confined to hospitalized patients but are increasingly encountered in outpatient pediatric populations. Integrating genomic resistance profiling with antimicrobial susceptibility testing provides valuable insights for resistance surveillance and supports evidence-based antibiotic selection. Continued monitoring of resistance mechanisms in community-acquired infections is essential to inform antimicrobial stewardship strategies and preserve the effectiveness of currently available antibiotics.

## Figures and Tables

**Table 1:** Culture results of the fecal samples.

Media	Macroscopic/Colony Morphology	Semiquantitative Interpretation *	Sample	Percentage
XLD Media	Yellowish	+1	15, 16, 17, 18	88.88%
		+2	3, 5, 9, 10, 13, 14	
		+4	1, 2, 6, 8, 11, 12	
EMB Media	Brownish	+4	1, 2, 5, 6, 12, 13	100%
		+1	7, 17, 18	
	Metallic Green	+4	3, 4, 6, 8,	
	Black	+1	16	
		+3	9, 10, 14	
	Metallic Brownish	+4	11	
	Smooth	+1	15	
Salmonella-Shigella	Pink	+1	16, 17, 18	38.89%
		+2	3, 5	
		+4	4, 6	
	Smooth	+1	15	
TCBS	Yellowish	+1	2, 4	16.67%
	Greenish	+2	8	
MacConkey	Pinkish	+1	7	27.78%
		+2	18	
		+3	17	
		+4	15, 16	

* Semiquantitative Interpretation +1: Scant growth; +2: Moderate growth; +3: Heavy growth; and +4: Very Heavy growth.

**Table 2:** Genomic characteristics of *Escherichia coli* and *Klebsiella pneumoniae* isolates.

Sample ID	Species	Sequence Type (ST)	Serotype	Phylogroup/Capsular Type
Sample 3	*E. coli*	ST-10	O153:H10	A
Sample 7	*E. coli*	ST-38	O7:H15	D
Sample 8	*E. coli*	ST-31	O25:H4	B2
Sample 9	*E. coli*	ST-10	O169:H45	A
Sample 14	*E. coli*	ST-167	O101:H10	A
Sample 15	*E. coli*	ST-69	O15:H18	D
Sample 16	*E. coli*	ST-1193	O75:H5	B2
Sample 18	*E. coli*	ST-6491	O127:H29	B1
Sample 1	*K. pneumoniae*	ST-231	KL51/O1	K2
Sample 2	*K. pneumoniae*	ST-101	KL17/O1	K17
Sample 4	*K. pneumoniae*	ST-15	KL38/O1	K24
Sample 5	*K. pneumoniae*	ST-4004	KL105/O1	K1
Sample 6	*K. pneumoniae*	ST-101	KL2/O1	K17
Sample 10	*K. pneumoniae*	ST-200	KL58/O3b	K1
Sample 11	*K. pneumoniae*	ST-45	KL24/O1	K24
Sample 12	*K. pneumoniae*	ST-23	KL43/O2a	K1
Sample 13	*K. pneumoniae*	ST-65	KL2/O1	K2
Sample 17	*K. pneumoniae*	ST-1758	KL27/O4	K5

**Table 3:** Correlation between antimicrobial resistance phenotype and genomic determinants.

Antibiotic Class	Antibiotic	Gene/Mutation	Resistant Isolates (%)	Non-Resistant Isolates (%)	*p*-Value
β-lactam (3rd gen cephalosporin)	Cefotaxime	*blaCTX-M-15*	High	Low	<0.05
β-lactam	Ceftazidime	*blaOXA-232*	Significantly higher	Lower	<0.05
Fluoroquinolone	Ciprofloxacin	*GyrA* (S83I/D87N)	Predominant	Rare/Absent	<0.01
Fluoroquinolone	Ciprofloxacin	*ParC* (S80I)	Predominant	Rare	<0.05
Fluoroquinolone	Ciprofloxacin	*qnrS1*	Moderate	Low	<0.05
Tetracycline	Doxycycline	*tet(A/B)*	Significantly higher	Lower	<0.05
Sulfonamide	Trimethoprim–sulfamethoxazole	*sul1/sul2*	Higher	Lower	<0.05
Carbapenem	Imipenem/Meropenem	*OmpK36* (T254S/E232R)	Enriched	Rare	<0.05
Multidrug	Multiple classes	*AcrR*/*RamR* mutations	MDR-dominant	Minimal	<0.01

**Table 4:** Plasmid replicon types and mobile genetic elements associated with resistance genes.

Isolate	Species	Key ARG(s)	Plasmid Replicon Type	Mobile Genetic Elements (MGE)
Sample 1	*K. pneumoniae*	*blaCTX-M-15*, *blaTEM-209*, *oqxA*	IncF	ISEcp1, Class 1 integron
Sample 2	*K. pneumoniae*	*blaCTX-M-15*, *blaOXA-232*, *fosA*	IncF, IncR	IS26, Class 1 integron
Sample 3	*E. coli*	*blaCTX-M-27*, *qnrS1*, *fosA6*	IncF	ISEcp1
Sample 4	*E. coli*	*blaOXA-1*, *tet(A)*, *sul2*	IncX	IS26
Sample 5	*K. pneumoniae*	*blaCTX-M-15*, *fosA*	IncHI	Class 1 integron
Sample 6	*K. pneumoniae*	*blaCTX-M-15*, *blaTEM-1B*	IncF	ISEcp1, IS26
Sample 7	*E. coli*	*blaOXA-1*, *mph(A)*, *tet(B)*	ND	ND
Sample 8	*E. coli*	*blaCTX-M-15*, *oqxA*	ND	ND
Sample 9	*E. coli*	*blaCTX-M-15*, *tet(A)*	ND	ND
Sample 10	*K. pneumoniae*	*blaCTX-M-15*, *oqxA*	IncF	IS26
Sample 11	*K. pneumoniae*	*blaTEM-1B*, *sul1*	ND	ND
Sample 12	*K. pneumoniae*	*blaTEM-209*, *fosA6*	ND	ND
Sample 13	*K. pneumoniae*	*blaCTX-M-15*, *oqxB*	IncF	ISEcp1
Sample 14	*E. coli*	*sul1*, *mph(A)*, *tet(A)*	ND	ND
Sample 15	*E. coli*	*oqxA*, *oqxB*	ND	ND
Sample 16	*E. coli*	*oqxA*	ND	ND
Sample 17	*K. pneumoniae*	*blaCTX-M-15*, *oqxA*	IncF	IS26
Sample 18	*E. coli*	*tet(A)*, *sul2*	ND	ND

ARG, antimicrobial resistance gene; ND, not detected; MGE, mobile genetic element.

## Data Availability

All data were available in this manuscript.

## Supplementary Materials

The following supporting information can be downloaded at: https://www.mdpi.com/article/10.3390/antibiotics15040331/s1, Figure S1. Culture Results in XLD Media. Figure S2. Culture Results in EMB Media. Figure S3. Culture Results in Salmonella-Shigella media. Figure S4. Culture Results in TCBS media. Figure S5. Culture Results in MacConkey media. Table S1. Samples FASTQ quality results before and after trimming. Table S2. Virulence genes and Pathotype. Table S3. Susceptibility Test compared to the genomic profile results.

## Abbreviations

The following abbreviations are used in this manuscript:

AMR	Antimicrobial Resistance;
APEC	Avian Pathogenic *Escherichia coli*;
ARG	Antimicrobial Resistance Gene;
AST	Antimicrobial Susceptibility Testing;
cgMLST	Core Genome Multilocus Sequence Typing;
CLSI	Clinical and Laboratory Standards Institute;
cKP	Classical *Klebsiella pneumoniae*;
DAEC	Diffusely Adherent *Escherichia coli*;
EAEC	Enteroaggregative *Escherichia coli*;
EMB	Eosin Methylene Blue;
EPEC	Enteropathogenic *Escherichia coli*;
ESBL	Extended-Spectrum β-Lactamase;
ETEC	Enterotoxigenic *Escherichia coli*;
ExPEC	Extraintestinal Pathogenic *Escherichia coli*;
hvKP	Hypervirulent *Klebsiella pneumoniae*;
IS	Insertion Sequence;
MDR	Multidrug Resistant;
MGE	Mobile Genetic Element;
MIC	Minimum Inhibitory Concentration;
MLST	Multilocus Sequence Typing;
ND	Not Detected;
QRDR	Quinolone Resistance–Determining Region;
SNP	Single Nucleotide Polymorphism;
SS	Salmonella–Shigella;
ST	Sequence Type;
TCBS	Thiosulfate Citrate Bile Salts Sucrose;
WGS	Whole-Genome Sequencing
