# Supplementary material for: Genomic Insights into Antimicrobial Resistance and Plasmid-Mediated Dissemination in Escherichia coli and Klebsiella pneumoniae from Pediatric Outpatients with Acute Diarrhea
Source: Antibiotics (Basel). 2026 Mar 25;15(4):331. doi: 10.3390/antibiotics15040331 (PMC13114209; doi:10.3390/antibiotics15040331)
Supplement: Supplementary file 1 [file antibiotics-15-00331-s001.zip › antibiotics-4118834-supplementary.pdf]

antibiotics

MDPI

Supplementary Materials

Table S1. Samples FASTQ quality results before and after trimming

Sample ID	Before Trimming		After Trimming		% reads with > Q30
	Sequence Count	Sequence length (bp)	Sequence Count	Sequence length (bp)	
Sample 1	1623613	35-151	1576315	35-151	90.00%
Sample 2	1703655	35-151	1668995	35-151	91.00%
Sample 3	1719544	35-151	1682767	35-151	90.00%
Sample 4	1881969	35-151	1856238	35-151	90.00%
Sample 5	1973571	35-151	1941329	35-151	92.00%
Sample 6	1761215	35-151	1738369	35-151	88.00%
Sample 7	2022942	35-151	1992586	35-151	90.00%
Sample 8	6180659	35-151	230081	35-151	92.00%
Sample 9	40962807	35-151	215436	35-151	85.00%
Sample 10	40206346	35-151	314889	35-151	88.00%
Sample 11	38981048	35-151	709969	35-151	90.00%
Sample 12	39860792	35-151	485076	35-151	91.00%
Sample 13	40801943	35-151	475684	35-151	89.00%
Sample 14	42630616	35-151	224093	35-151	90.00%
Sample 15	21452942	35-151	146166	35-151	93.00%
Sample 16	37861687	35-151	257229	35-151	94.00%
Sample 17	18995440	35-151	976544	35-151	89.00%
Sample 18	42789617	35-151	285384	35-151	92.00%

Table S2. Virulence genes and Pathotype

Gene	Gene Description	Pathotype
irp1	Iron-regulated outer membrane protein 1	ExPEC
irp2	Iron-regulated outer membrane protein 2	ExPEC
ybtSXQPA	Yersiniabactin biosynthesis genes	ExPEC
fyuA	Yersiniabactin receptor	ExPEC
iutA	Aerobactin receptor	ExPEC
ybt	Yersiniabactin biosynthesis regulator	ExPEC
Aap	Adhesin AIDA-I precursor	APEC, UPEC
air	Adhesin involved in biofilm formation	UPEC
agg3B, agg3C, agg3D	Aggregative adherence fimbriae subunits	EAEC
afaA, afaB	Dr family adhesins	DAEC
astA	Heat-stable enterotoxin	ETEC
traT	Serum resistance protein	ExPEC
iss	Increased serum survival protein	ExPEC
chuA	Heme receptor	ExPEC
sat	Secreted autotransporter toxin	UPEC
terC	Tellurium resistance protein	ExPEC
eilA	Iron uptake regulation protein	ExPEC
nfaE	Non-fimbrial adhesin E	ExPEC
kpsE	Capsule export protein	ExPEC
kpsMII	Capsule biosynthesis protein	ExPEC
capU	Capsule biosynthesis protein	ExPEC
AslA	Antisense RNA regulating biofilm formation	UPEC
csgA	Curli fimbriae subunit	ExPEC
fdeC	Fimbrial adhesin	ExPEC
fimH	Type 1 fimbrial adhesin	ExPEC, UPEC
iha	Iron-regulated adhesin	ExPEC
ireA	Iron-regulated outer membrane protein	ExPEC
yehD	Outer membrane protein	ExPEC
hlyA, hlyB, hlyC, hlyD, hlyE	Hemolysin proteins	ExPEC, UPEC
clbA, clbB	Colibactin biosynthesis proteins	ExPEC
iucC	Aerobactin biosynthesis protein	ExPEC
uge	UDP-glucose 4-epimerase	ExPEC
magA	Mucoviscosity-associated gene A	ExPEC
rmpC	Regulator of mucoid phenotype C	ExPEC
evgA	Two-component system regulator	ExPEC
ycfM	Outer membrane protein	ExPEC

pgaABCD	Poly-β-1,6-N-acetylglucosamine biosynthesis genes	ExPEC
bap	Biofilm-associated protein	ExPEC
mrkA	Type 3 fimbrial adhesin	ExPEC, UPEC
nlpI	Outer membrane lipoprotein	ExPEC
pet	Plasmid-encoded toxin	EAEC
cea	Colicin E1 immunity protein	ExPEC
colE5	Colicin E5	ExPEC
clpK1	ATP-dependent protease Clp protease	ExPEC
yehA, yehB, yehC	Outer membrane proteins	ExPEC
eatA	Secreted autotransporter toxin	ETEC
gad	Glutamate decarboxylase system	ExPEC
lpfA	Long polar fimbriae subunit	ExPEC
Pic	Secreted autotransporter toxin	ExPEC
SfaS	S-fimbrial adhesin	ExPEC
PapG, PapC, PapE, PapF, PapH, PapI, PapJ, PapK, PapX, PapY, PapZ	P fimbrial adhesin subunits	ExPEC
AfaD	Afimbrial adhesin D	DAEC
FimH	Type 1 fimbrial adhesin	ExPEC, UPEC
AfaE	Afimbrial adhesin E	DAEC
EspY2	Type VI secretion system effector	ExPEC
HlyF	Hemolysin F	ExPEC
	**Klebsiella pneumoniae**	
bap	Biofilm-associated protein Bap	cKp
capU	Capsular polysaccharide biosynthesis protein CapU	hvKp
clbA, clbB	Colibactin biosynthesis proteins ClbA and ClbB	hvKp, cKp
entABCDEF	Enterobactin biosynthesis proteins EntA, EntB, EntC, EntD, EntE, and EntF	hvKp
fepA	Ferric enterobactin transport protein FepA	hvKp
fhuC, fhuB, fhuA, fhuD	Ferric hydroxamate receptor proteins FhuC, FhuB, FhuA, and FhuD	cKp
fimC, fimD	Chaperone proteins FimC and FimD	cKp
FimH-1, fimA, fimE	Type 1 fimbrial proteins FimH-1, FimA, and FimE	hvKp, cKp
fyuA, iutA, iucA, iroB	Siderophore receptor and biosynthesis proteins FyuA, IutA, IucA, and IroB	hvKp
galF	UTP-glucose-1-phosphate uridylyltransferase GalF	hvKp
hlyA, hlyB, hlyC, hlyD	Hemolysins HlyA, HlyB, HlyC, and HlyD	hvKp
kfuABC	Ferric iron uptake system proteins KfuA, KfuB, and KfuC	hvKp
kpsM, kpsT	Capsular polysaccharide transport proteins KpsM and KpsT	cKp

kpsS, kpsE, kpsF	Capsular polysaccharide transport and export proteins KpsS, KpsE, and KpsF	hvKp
lrp-1	Leucine-responsive regulatory protein Lrp-1	cKp
magA, uge	Outer membrane protein MagA and UDP-glucose 4-epimerase Uge	hvKp
manC	Mannose-1-phosphate guanylyltransferase ManC	cKp
mrkD, mrkB, mrkE, mrkA	Fimbrial assembly proteins MrkD, MrkB, MrkE, and MrkA	hvKp, cKp
nfaE	N-acetylmuramoyl-L-alanine amidase NfaE	cKp
ompA, pilA	Outer membrane protein OmpA and Pilus assembly protein PilA	hvKp
pgaABCD, bap	Biofilm formation proteins PgaABCD and Bap	cKp
pilV	Pilus assembly protein PilV	cKp
RmpA2, RmpA, RcsAB, RcsC, RcsD	Regulators of mucoid phenotype and response regulators RmpA2, RmpA, RcsAB, RcsC, and RcsD	hvKp
rmpC	Regulator of mucoid phenotype C	hvKp
wza, wzb, wzc, wcaG, wcaJ	Capsule export and O-antigen biosynthesis proteins Wza, Wzb, Wzc, WcaG, and WcaJ	hvKp
wzm	Lipopolysaccharide export system protein Wzm	cKp
ybtS, ybtAEPQSTUX	Yersiniabactin biosynthetic proteins YbtS, YbtAEPQSTUX	hvKp
ycfM	Hypothetical protein YcfM	cKp

Table S3. Susceptibility Test compared to the genomic profile results

		**Susceptibility Test**					**Genomic Profile**				
		**Ampicillin**	**Trimetho-Prime**	**Ceftazedime**	**Ciprofloxcin**	**Piperacillin-Tazobactam**	**Ampicillin**	**Trimetho -Prime**	**Ceftazedime**	**Ciproflo -xcin**	**Piperacillin-Tazobactam**
K.P	Sample 1	R	S	R	R	R	R *(blaCTX-M-15 etc.)*	R *(OqxA,B)*	R *(blaCTX-M-15)*	R *(OqxA)*	R *(blaCTX-M-15)*
	Sample 2	R	R	R	R	R	R *(blaSHV-5 etc.)*	R *(OqxA,B)*	R *(blaCTX-M-15)*	R *(OqxA)*	R *(blaCTX-M-15)*
	Sample 4	R	S	S	S	S	R *(blaOXA-232 etc.)*	R *(OqxA,B)*	S	R *(OqxA)*	R *(blaOXA-232)*
	Sample 5	R	R	S	R	S	R *(blaSHV-62 etc.)*	R *(OqxA,B)*	R *(blaACT-16)*	R *(OqxA)*	R *(blaCTX-M-15)*
	Sample 6	R	S	S	S	S	R *(blaCTX-M-15 etc.)*	R *(OqxA,B)*	R *(blaCFE-1)*	R *(OqxA)*	R *(blaTEM-164)*
	Sample 10	S	S	S	S	S	S	R *(OqxA,B)*	S	R *(OqxA)*	S
	Sample 11	R	S	S	S	S	S	R *(dfrA14)*	S	R *(OqxB)*	S
	Sample 12	R	S	S	S	S	R *(blaOXA-232 etc.)*	R *(OqxA,B)*	S	R *(OqxA)*	R *(blaOXA-232)*
	Sample 13	R	R	R	S	R	S	R *(OqxB)*	S	R *(OqxA)*	S
	Sample 17	R	S	S	S	R	S	R *(OqxB)*	S	R *(OqxA)*	S
E. COLI	Sample 3	R	R	S	S	S	R *(blaCTX-M-27)*	S	R *(blaCTX-M-27)*	S	S
	Sample 7	R	S	S	S	S	R *(blaOXA-10 etc.)*	S	S	R *(qnrS1)*	R *(blaOXA-10)*

Sample 8	R	R	S	R	S	R *(blaCTX-M-15)*	R *(OqxA,B)*	R *(blaCTX-M-15)*	R *(OqxA)*	S
Sample 9	S	S	S	S	S	R *(blaCTX-M-15)*	R *(OqxA,B)*	R *(blaCTX-M-15)*	R *(OqxA)*	S
Sample 14	S	R	S	S	S	S	R *(dfrA14)*	S	S	S
Sample 15	R	R	S	S	S	S	R *(OqxA,B)*	S	R *(OqxA,B)*	S
Sample 16	R	S	S	S	S	S	R *(OqxA,B)*	S	R *(OqxA,B)*	S
Sample 18	R	S	S	S	S	S	R *(dfrA12)*	S	S	S

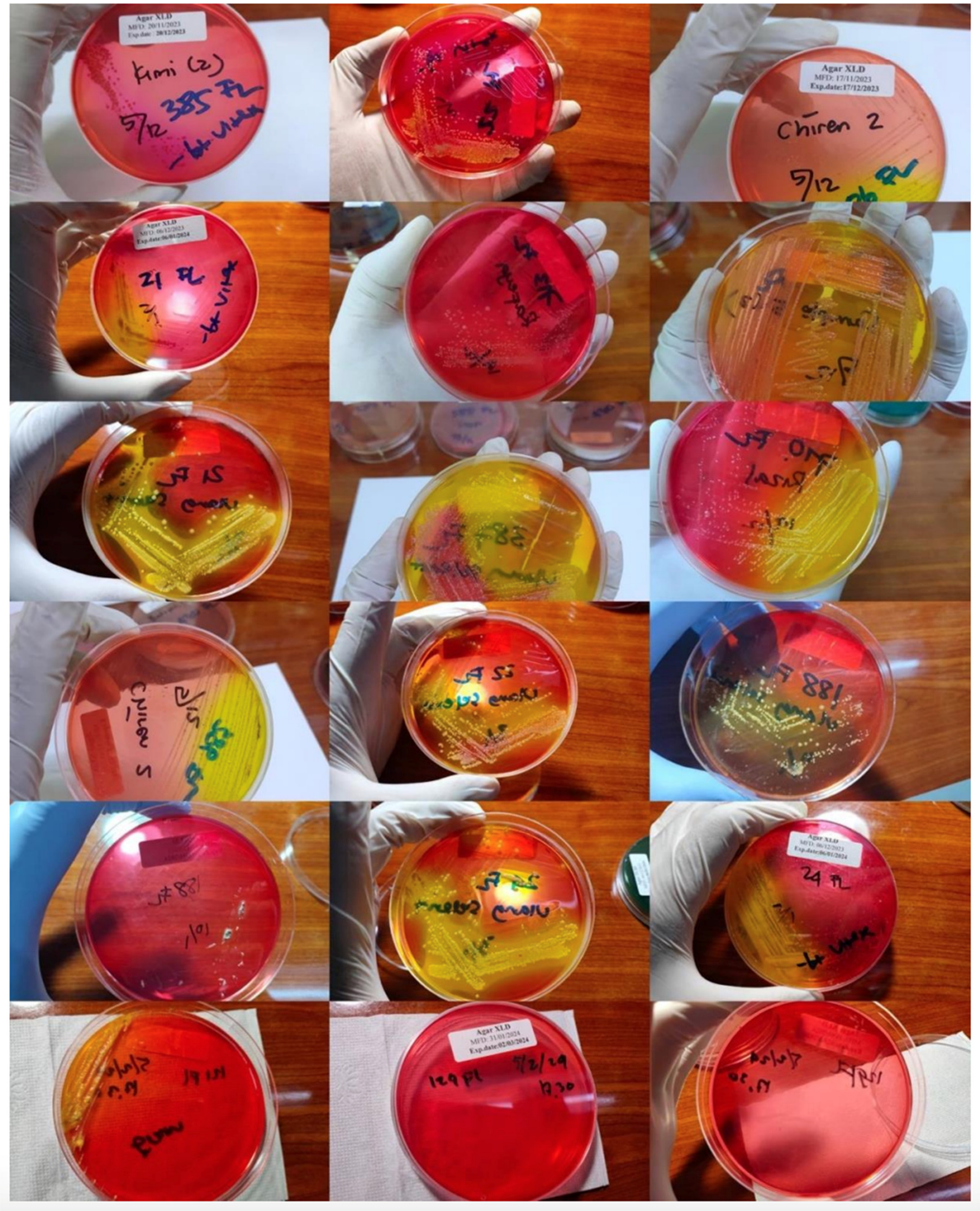

Figure S1. Culture Results in XLD Media

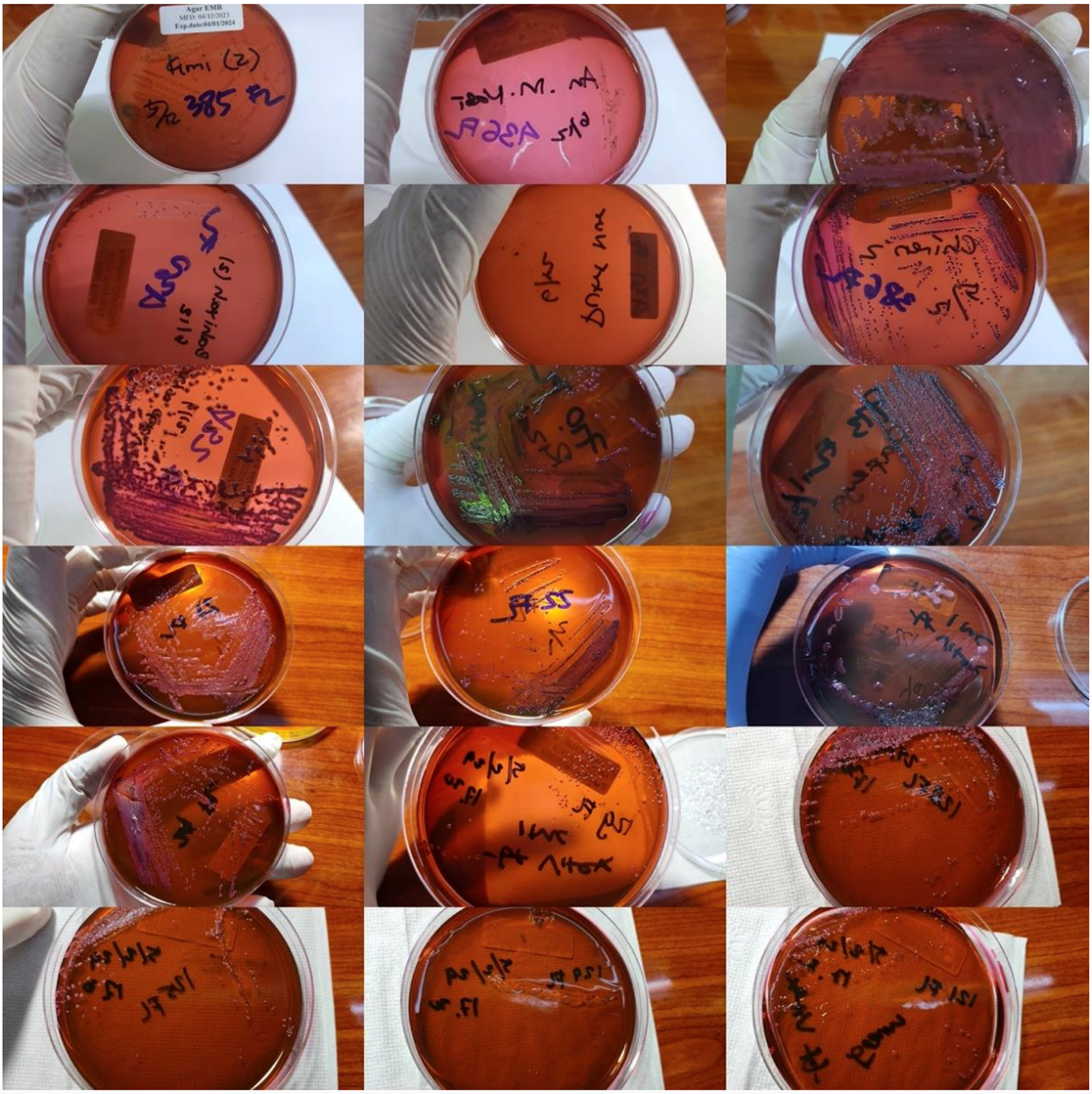

Figure S2. Culture Results in EMB Media

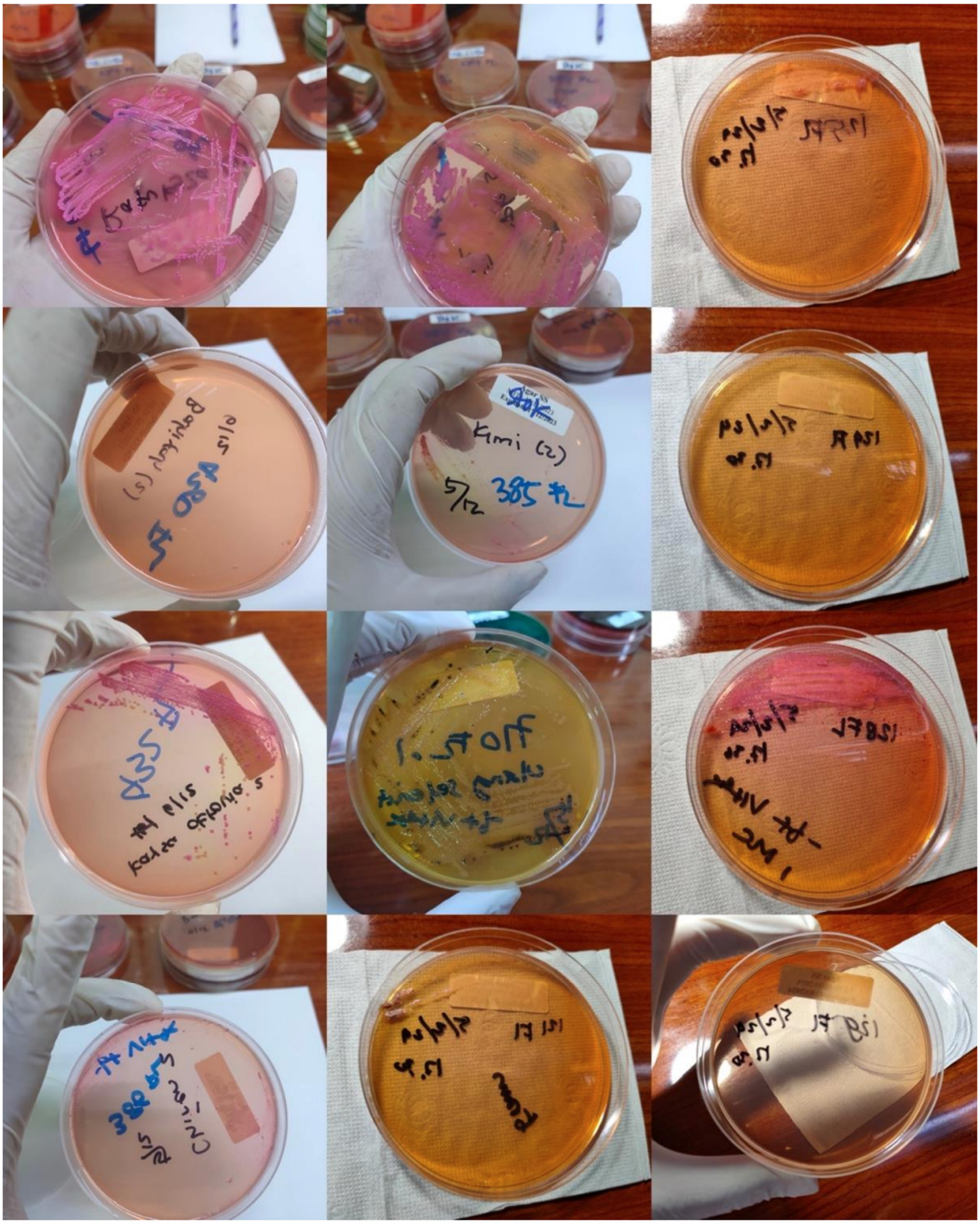

Figure S3. Culture Results in Salmonella-Shigella media

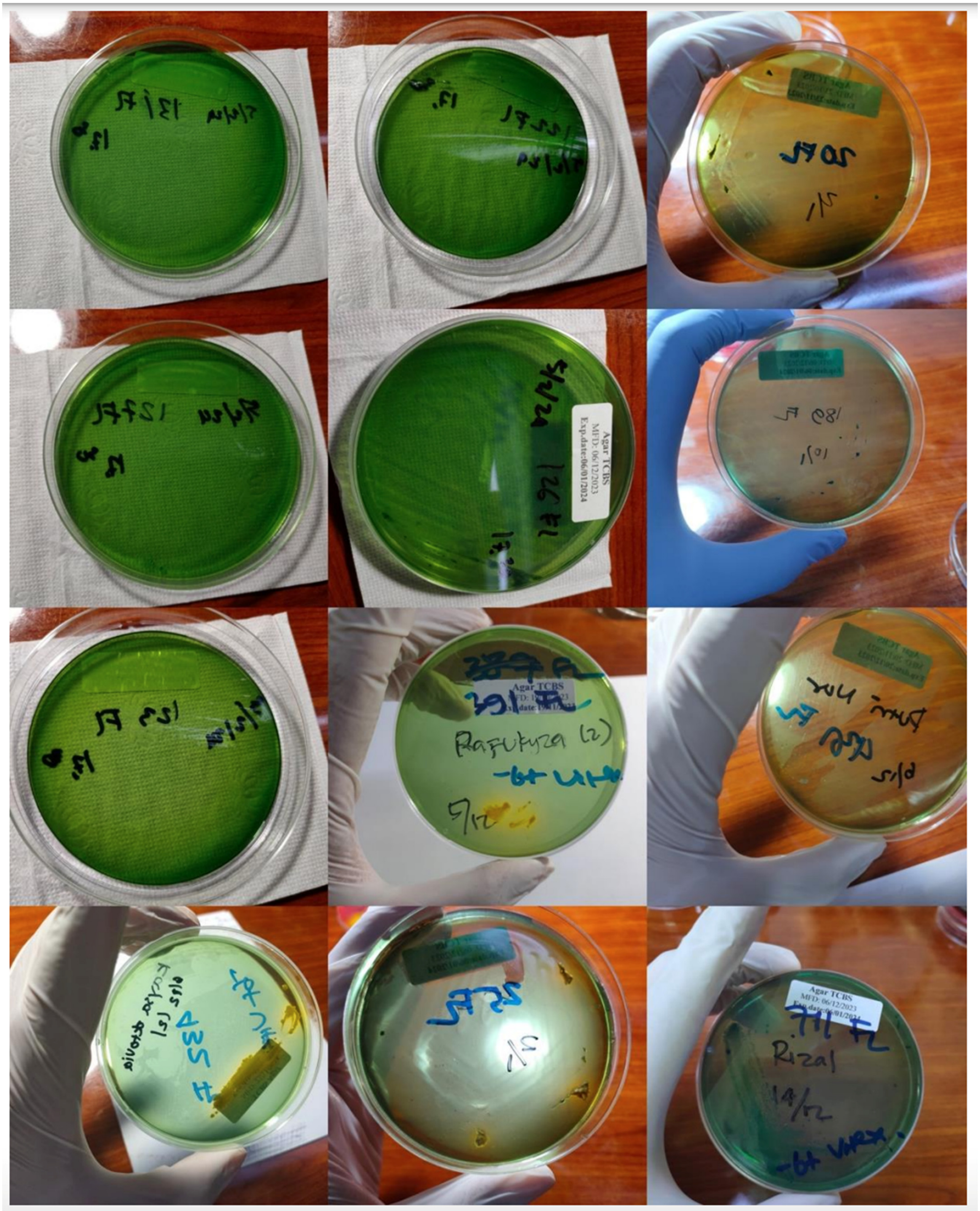

Figure S4. Culture Results in TCBS media

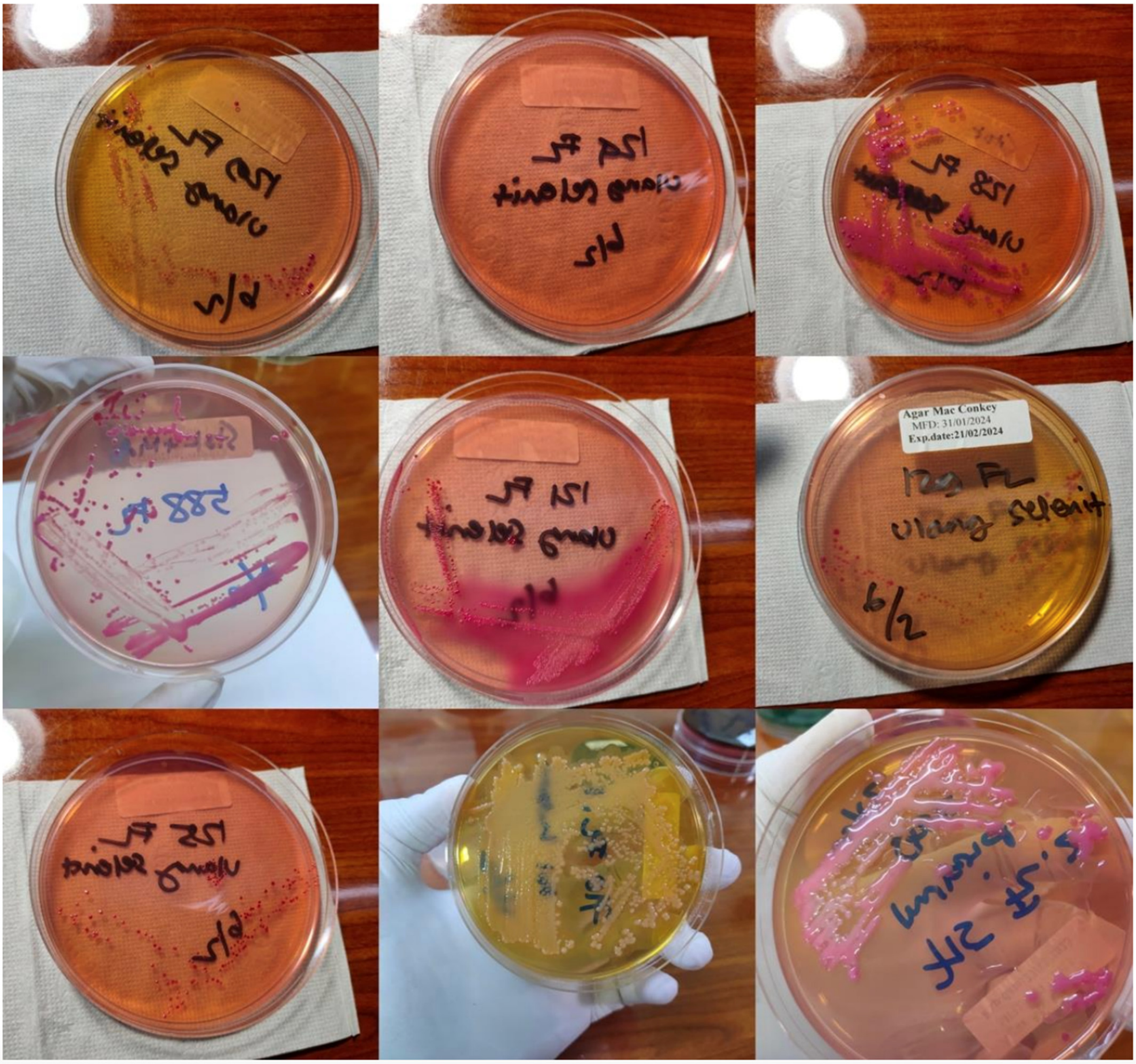

Figure S5. Culture Results in MacConkey media

Disclaimer/Publisher's Note: The statements, opinions and data contained in all publications are solely those of the individual author(s) and contributor(s) and not of MDPI and/or the editor(s). MDPI and/or the editor(s) disclaim responsibility for any injury to people or property resulting from any ideas, methods, instructions or products referred to in the content.
